# Breastfeeding Practices and Determinant Factors of Exclusive Breastfeeding among Mothers of Children Aged 0–23 Months in Northwestern Romania

**DOI:** 10.3390/nu13113998

**Published:** 2021-11-10

**Authors:** Anamaria Cozma-Petruţ, Lorena Filip, Roxana Banc, Oana Mîrza, Laura Gavrilaş, Daniela Ciobârcă, Ioana Badiu-Tişa, Simona Codruţa Hegheş, Cristian Olimpiu Popa, Doina Miere

**Affiliations:** 1 Department of Bromatology, Hygiene, Nutrition, “Iuliu Haţieganu” University of Medicine and Pharmacy, 6 Pasteur Street, 400349 Cluj-Napoca, Romania; anamaria.cozma@umfcluj.ro (A.C.-P.); lfilip@umfcluj.ro (L.F.); oana.stanciu@umfcluj.ro (O.M.); laura.gavrilas@umfcluj.ro (L.G.); muresan.daniela@umfcluj.ro (D.C.); dmiere@umfcluj.ro (D.M.); 2 Department of Mother and Child Care, “Iuliu Haţieganu” University of Medicine and Pharmacy, 2–4 Câmpeni Street, 400217 Cluj-Napoca, Romania; badiu.doina@umfcluj.ro; 3 Department of Pharmaceutical Analysis, “Iuliu Haţieganu” University of Medicine and Pharmacy, 6 Pasteur Street, 400349 Cluj-Napoca, Romania; cmaier@umfcluj.ro; 4 Department of European Studies, Babeș-Bolyai University, 1 E. de Martonne Street, 400090 Cluj-Napoca, Romania; cris.popa@gmail.com

**Keywords:** breastfeeding practices, exclusive breastfeeding, determinants, Romania

## Abstract

In terms of breastfeeding (BF) practices in Romania, there is a lack of up-to-date data. The aim of the present study was to assess current BF practices, and to investigate the factors associated with exclusive BF (EBF) under 6 months of age in northwestern Romania. A structured questionnaire was used to collect data among 1399 mothers of children aged 0–23 months, recruited between March and June 2019, from the community. BF practices were evaluated based on the World Health Organization indicators for assessing infant and young child feeding practices, whereas determinants of EBF were explored using logistic regression models. Almost all mothers (95.7%) breastfed their child at least once. The EBF rate was 46.7%, the continued BF rate at one year of age was 54.2%, and the continued BF rate at 2 years of age was 30.3%. The place of delivery and parental leave duration were strong determinants of EBF. The improving rates observed in this study for all the BF practices assessed suggest the continuation of efforts to develop effective national policies and programs for promoting, protecting, and supporting BF in Romania. Particular emphasis should be given to the creation in maternity hospitals of an environment that is supportive towards breastfeeding.

## 1. Introduction

Breastfeeding is beneficial for both child and mother. The maternal benefits of breastfeeding include a more rapid postpartum involution and return to pre-pregnancy weight, as well as a reduced risk of breast, ovarian, and endometrial cancer [1]. Breastfeeding also strengthens the mother-child bond, and improves household productivity by eliminating the expenses of purchasing formula [1,2]. Furthermore, breast milk represents the best source of nutrition for infants [3]. Breastfeeding not only supports healthy growth and development and reduces the risk of infectious diseases in infancy, but also decreases the risk of obesity in childhood and its associated metabolic diseases in later years [4,5,6]. Therefore, in European countries, including Romania, where excess body weight in children currently poses a significant burden, optimal breastfeeding practices could have an essential role in the prevention of obesity and its adverse consequences [5,7,8,9,10].

The World Health Organization (WHO) recommends the practice of exclusive breastfeeding (EBF) for the first 6 months of life, followed by the gradual introduction of adequate solid foods and continuation of breastfeeding for up to 2 years of age or beyond [11]. Nevertheless, within the WHO European Region, breastfeeding practices do not meet the above-mentioned WHO recommendations [12]. Moreover, EBF practice in Europe does not reach the 2025 World Health Assembly’s Global Target for Nutrition that aims to increase the rate of EBF in the first six months up to at least 50% [13]. A report published in 2020 showed that the rate of EBF for infants under 6 months of age was 40% in the 16 European countries that provided data in this regard [12]. However, the report included no information for Romania.

Indeed, there are little up-to-date data available on breastfeeding practices in Romania. According to a survey published in 2016, Romania ranks among the WHO European Region Member States with the lowest rates in terms of breastfeeding practices [9]. The reported estimates, which originate from the 2004 Reproductive Health Survey conducted by the Romanian Ministry of Health, indicated that only 15.8% of infants were exclusively breastfed in the first 6 months [14]. Furthermore, the latest 2011 National Infant Feeding Survey suggested a decline in breastfeeding practices in Romania, with a rate of EBF under 6 months of age of only 12.6% [15]. The same survey reported a rate of continued breastfeeding at 1 year as low as 21.3% [15]. Such suboptimal rates for breastfeeding practices should be thoroughly investigated. In particular, the identification of modifiable factors affecting EBF practice is important. In fact, a growing body of evidence shows that breastfeeding practices are influenced by a range of sociodemographic factors (i.e., maternal age, marital status, education level, employment status, income) and pre/perinatal factors (i.e., parity, prenatal classes, mode of delivery, early skin-to-skin contact practice, early initiation of breastfeeding, rooming-in practice) [1,16,17,18]. Thus, the aim of the present study was to assess the current breastfeeding practices and to investigate the sociodemographic and pre/perinatal factors associated with EBF in northwestern Romania.

## 2. Materials and Methods

### 2.1. Study Area

This study was conducted in the counties of Bihor, Bistrița-Năsăud, Cluj, Maramureș, Satu-Mare, and Sălaj, which belong to the northwestern development region of Romania. Representing 14.3% of the country’s territory and 13.11% of the total population, the northwestern region displays a positive economic status, contributing at 12.23% the national Gross Domestic Product in 2017, ranking second nationally. The unemployment rate in the region was 3.7% in 2017, lower than the national level [19].

As of 2018, the region was distinguished by a birth rate of 9.3 live births per thousand inhabitants, which was above the national rate of 8.6 [19]. In 2019, the year in which the study was conducted, northwestern Romania had a population of 591,510 women of fertile age (15 to 49 years), who were living to a greater extent in the urban areas (55.3%) than in the rural ones (44.7%) [20]. Little is known regarding the current educational level of this female population. The latest data are provided by the 2011 census, reporting that among women aged 15–49 years in northwestern Romania, 19.3% had a bachelor’s degree or higher, and 73.7% completed high school or equivalent, whereas 7% had at most a secondary school level [21].

### 2.2. Participants and Data Collection

**Figure 1:**
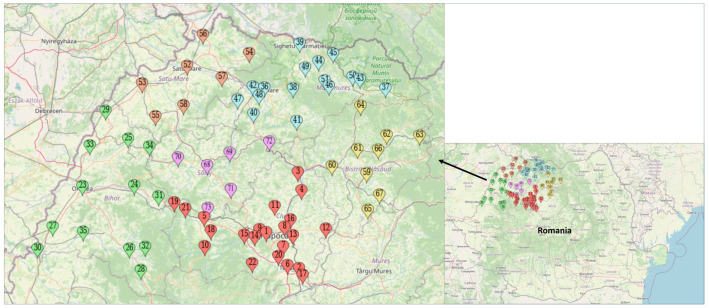
Geographical distribution of the sample, according to county, in northwestern Romania. The map was created with the Leaflet/MapCustomizer program, version 1.7.1. Legend: red dots (no. 1–22)—Cluj County; green dots (no. 23–35)—Bihor county; blue dots (no. 36–51)—Maramureș county; orange dots (no. 52–58)—Satu Mare county; yellow dots (no. 59–67)—Bistrița-Năsăud county; purple dots (no. 68–73)—Sălaj county.

In this cross-sectional study, mothers of children aged 0–23 months were recruited by promoting the survey in child health centers, nurseries, and playgrounds spread across the urban and rural areas of northwestern Romania. Figure 1 displays the geographical distribution of the sample. All data were collected between March and June 2019. Eligible mothers were provided with proper oral and written information about the study while attending the child health center, nursery, or playground. In total, 1399 consenting mothers who met the inclusion criteria were interviewed by trained data collectors, using a structured questionnaire. Details about the study design and sampling have been previously described [22].

### 2.3. Study Instrument

A pre-tested questionnaire included questions about the sociodemographic and pre/perinatal characteristics of the mothers, followed by questions concerning infant feeding practices. More information about the study methodology has been published elsewhere [22].

Breastfeeding practices were evaluated using the WHO indicators for assessing infant and young child feeding practices [23]. Indicators determined in the present study and their definitions are as follows:

- Exclusive breastfeeding under 6 months (EBF): the proportion of infants 0–5 months of age who were fed exclusively with breast milk in the last 24 h;
- Continued breastfeeding at 1 year: the proportion of children 12–15 months of age who were fed breast milk in the last 24 h;
- Continued breastfeeding at 2 years: the proportion of children 20–23 months of age who were fed breast milk in the last 24 h;
- Children ever breastfed: the proportion of children born in the last 24 months who were ever breastfed.

### 2.4. Statistical Analysis

Data analysis was carried out using STATA version 16 (StataCorp. 2019. Stata Statistical Software: Release 16. StataCorp LLC, College Station, TX, USA). Apart from descriptive statistics, simple (univariate) logistic regression analysis was conducted to determine the strength of association between each independent variable (sociodemographic characteristics, pre/perinatal characteristics) and the indicator EBF. Variables that were significant at *p*-value of less than 0.05 at the univariate logistic analysis were included in the multiple regression model. Adjusted odds ratios (AOR) of significantly associated variables and their corresponding 95% confidence intervals (CI) have been reported. In all the statistical analyses, a *p*-value of less than 0.05 was considered significant.

### 2.5. Ethics

The study was approved by the Research Ethics Committee of the “Iuliu Hațieganu” University of Medicine and Pharmacy Cluj-Napoca, Romania (Approval no. 74/11.03.2019), and conducted in compliance with the guidelines of the Declaration of Helsinki. Written informed consent was obtained from all mothers who participates in the study. Processing of participants data was in agreement with the Privacy Act (General Data Protection Regulation—GDPR, EU Regulation 2016/679).

## 3. Results

### 3.1. Characteristics of the Participants

The characteristics of the mothers involved in this study (*n* = 1399) are summarized in Table 1. The majority were aged 25 to 34 years (67.2%), and had a bachelor’s degree or higher (69.8%). Almost all mothers (98.8%) were married or living with a partner, and the majority resided in urban areas (73.4%). With regard to employment status, 88% were employed, but only 12.8% reported an excellent family financial wellbeing.

The mothers mostly delivered at public hospitals (73%), and more than half (51.5%) delivered their child by caesarean section. For 59.5% of mothers, this was their first child. Regarding infant gender, 51.2% of the children were males, and 48.8% of the children were females. At the time of the interview, 27% of the children were below 6 months old, 29% of the children were 6–11 months old, and 44% were 12–23 months old.

Almost one third of the mothers (28.6%) had taken a prenatal birth and childcare class, and half received breastfeeding education during prenatal visits. The majority of the mothers (75.2%) received postnatal breastfeeding education provided by a healthcare professional. Furthermore, only a low proportion of the mothers (21.7%) had skin-to-skin contact with their newborn during the first hour after birth. Likewise, only a few mothers (24.3%) initiated breastfeeding the first hour after birth.

### 3.2. Breastfeeding Practices

Table 2 presents the breastfeeding practices among the study participants. Almost all of the mothers (95.7%) breastfed their child at least once. The EBF rate was 46.7%. Moreover, the rate of continued breastfeeding at 1 year was 54.2%, and the rate of continued breastfeeding at 2 years was 30.3%.

### 3.3. Determinants of Exclusive Breastfeeding

Table 3 presents the unadjusted odds ratio (OR) and adjusted odds ratio (AOR) at a 95% confidence interval (CI) of sociodemographic and pre/perinatal factors associated with EBF. In the unadjusted model, factors associated with EBF included maternal age, maternal education, family financial wellbeing, duration of parental leave, mode of delivery, place of delivery, practice of skin-to-skin contact during the first hour after birth, and initiation of breastfeeding during the first hour after birth.

In the adjusted model, the place of delivery and the duration of parental leave showed statistically significant associations with EBF. Mothers who gave birth at a private hospital were more likely to practice EBF, compared to those who gave birth at a public hospital (AOR 1.62, 95% CI 1.06, 2.48; *p* = 0.026). Likewise, mothers who opted to return to work when the child was 22 months of age or older were more likely to practice EBF, compared to those who opted to return to work when the child was less than 22 months of age (AOR 7.90, 95% CI 3.43, 18.22; *p* = 0.000).

## 4. Discussion

Acknowledging the numerous short-term and long-term health benefits of breastfeeding, the present study was aimed at assessing breastfeeding practices and examining sociodemographic and pre/perinatal determinants of EBF in northwestern Romania.

The EBF rate among the mothers involved in the study was 46.7%. This rate in northwestern Romania is substantially higher than the rate of 12.6% reported at national level by the 2011 Romanian Infant Feeding Survey [15], suggesting a significant improvement in EBF practice in the last decade. It should be noted that this latter survey and the present study are suitable for comparison in terms of definitions used, because both have evaluated breastfeeding practices using the 2010 WHO indicators for assessing infant and young child feeding practices [23].

The EBF rate found in this study, in northwestern Romania, is also higher than the median estimate of 40% for WHO European Region countries [12]. More specifically, the EBF rate is higher than that in some other European countries, such as France (10%), Germany (12%), the UK (17%), Ukraine (20%), Spain (29%), or the Republic of Moldova (36%), but remains lower than the EBF rate in Croatia (65%) [24]. Likewise, the EBF rate in this study is close to the minimum target of 50% endorsed by the World Health Assembly for 2025 [13].

When comparing the findings of this study with those of the 2011 Romanian Infant Feeding Survey, a positive evolution was observed not only in EBF rate, but also in the rates of other breastfeeding practices [15]. The rate of continued breastfeeding at 1 year was 54.2%, more than double compared to 2011 (21.3%) [15]. Likewise, the rate of the indicator “children ever breastfed” was 95.7%, slightly higher compared to 2011 (93.0%) [15]. Similarly, over the last years, an improvement in breastfeeding practices has been observed in other European countries, such as Croatia [25], Bulgaria [26], Greece [17], France [27], and England [28].

The rates of breastfeeding practices in the current study show values that are encouraging, especially when considering the benefits that an optimal breastfeeding could provide to the health and nutrition status of infants and young children in Romania. The practice of EBF up to 6 months and the continuation of breastfeeding combined with complementary foods from the age of 6 months represent optimal practices for the prevention of childhood obesity, which is currently an important health issue in Romania [7,8,10,29]. In particular, EBF prevents the early introduction of solid foods, considered before 4 months of age, a practice that has been associated with an increased risk for excessive weight gain in childhood [5,30]. Moreover, breastfeeding seems to improve the acceptance of complementary foods, especially fruits and vegetables, upon their introduction in the child’s diet, thus favoring a health-promoting dietary diversity [31]. Although the positive effects of breastfeeding are unquestionable, it is also worth mentioning that it may increase the risk of iron deficiency among older breastfed infants [32]. This topic is of particular interest for Romania, where iron deficiency anemia in children aged 6 to 23 months old still represents a significant health issue [33]. Considering that at approximately 6 months of age, breastfed infants start relying on iron-rich solid foods to meet their iron needs [34], future studies should aim at assessing the adequacy of complementary feeding patterns in Romania.

Furthermore, the present study showed that the place of delivery is a strong determinant of EBF, with mothers who delivered at a private maternity hospital being more likely to exclusively breastfeed up to 6 months, compared to mothers who delivered at a public maternity hospital. This is contrary to what the evidence from other European countries shows about the influence of the place of delivery on EBF practice [35,36,37]. In fact, a recent study conducted in Italy indicated that a private health facility, although providing 100% rooming-in and hotel level comforts, registered lower rates of EBF than several public health facilities [37]. However, the same study reported no differences between the different types of hospitals in the information about infant care and breastfeeding that the mothers received from healthcare professionals [37]. This may not be the case in the present study, in which the positive association between EBF and private sector deliveries could be explained by potentially higher quality medical services provided by private hospitals in Romania. According to Coman et al. [38], private care in Romania is characterized by a better interaction between the medical staff and the patient, and by a greater attention that healthcare professionals pay to the patients’ needs. When it comes to private maternity hospitals, they usually ensure the presence of a lactation consultant and the intervention of a psychologist, who support mothers in the postnatal period to initiate the care and breastfeeding of the child [39]. Indeed, it has been described in the literature that the more support mothers receive from maternity staff during the first days after childbirth, the better the likelihood for exclusive prolonged breastfeeding [37,40].

The present study also showed that the rate of EBF was higher among mothers who opted to return to work at 22 months or more post-childbirth than among those who opted to return to work at less than 22 months post-childbirth. These results are consistent with findings of earlier studies, which suggested that the time between childbirth and return to work is a predictor of EBF duration [41,42,43,44]. Indeed, the Romanian parental leave policy shows features that may support breastfeeding among working mothers. In Romania, there are 126 days of maternity leave, which are compensated at 85% of the mean monthly gross income obtained in the last six months before maternity leave. Mothers can take a maximum of 63 days before birth, and the remaining 63 days after birth, or the entire period of 126 days after the birth. It is mandatory to take at least 42 days of post-natal leave. Following this, mothers and fathers are entitled to parental leave until the child is 24 months of age, which is paid at 85% of earnings over the last 12 months. At least one month from the total parental leave available is granted to the parent who is eligible for leave, but has not requested the right to leave. Usually, this parent is the father, leaving the mother the opportunity to care for the child until he turns 23 months old. However, a labor market insertion incentive is given until the child is 36 months of age to the parent taking parental leave, if the parent returns to work at least 60 days before the child is 24 months old. In this context, many mothers choose to return to work at 22 months from childbirth [45].

Besides the factors that have been taken into account for in the multivariable analysis, other changes have occurred in Romania during the past years, which also may have contributed to the increasing trend in breastfeeding practices rates. Since 2008, during World Breastfeeding Week, the National Center for Health Assessment and Promotion organizes every year a national breastfeeding awareness campaign and educational activities to promote breastfeeding among parents and health professionals [46]. In 2016, the National Institute of Public Health published a prevention guide containing the national recommendations for optimal nutrition in the first 1000 days of life. The guide, addressed to health professionals, pregnant women, and lactating women, presents the benefits of breastfeeding, recommending EBF for the first 6 months of age, and continuation of breastfeeding combined with solid foods up to 24 months and beyond [47]. Moreover, in 2016, through the initiative of several non-governmental organizations and medical societies, the vote was obtained from the National Audiovisual Council to initiate the broadcast on all TV and radio stations of the message “exclusively breastfeeding the baby in the first six months is essential for a healthy life” [48].

In the context of the actions undertaken to promote breastfeeding in Romania, it is also worth mentioning the measures for the implementation of the WHO/UNICEF Baby-Friendly Hospital Initiative (BFHI). Out of the 182 maternity hospitals in Romania, 32 have been accredited as baby-friendly by 2013 [49]. These data show some progress, but indicate at the same time that more efforts must be directed for every maternity hospital in Romania to adopt the BFHI principles. Similar perspectives are provided by the present study, with focus on early skin-to-skin contact and early breastfeeding initiation as components of the BFHI “Ten Steps to Successful Breastfeeding”. The study reported that both the rate of skin-to-skin contact during the first hour after birth (21.7%) and the rate of initiation of breastfeeding during the first hour after birth (24.3%) were suboptimal among the study participants (*n* = 1399), thereby suggesting the need for a more breastfeeding supportive environment in the maternity hospitals in Romania.

As with all studies, the present study has some limitations that must be addressed. Asking mothers to recall aspects regarding their breastfeeding practices for a time period of up to two years ago is an important limitation. Nevertheless, according to recent research, maternal recall represents the standard in the case of large epidemiological surveys [50]. Likewise, a two-year recall period is the standard recommended by the WHO [23].

The recruitment of participants from child health centers, nurseries, and playgrounds is the next limitation of the study, in that it potentially affected the representativeness of the current sample compared to the general population of mothers of children aged 0–23 months that reside in northwestern Romania. It can be assumed an underrepresentation of mothers who live in rural areas, as well as an overrepresentation of mothers having tertiary education. Yet, the statistical analysis revealed that EBF practice was not different between mothers, in relation to their place of residence and educational level.

Another limitation of this study concerns the fact that the sample was drawn from a single region in Romania, and that, for this region there is little updated information on its sociodemographic characteristics compared to those in the whole country. Therefore, the findings of the current study may not be generalizable to Romania as a whole. Future studies are required to assess breastfeeding practices and their associated factors in all regions of Romania.

Despite its methodological limitations, the present study also has several strengths. The study includes a large sample size that confers an adequate statistical power. Even more important, the study contributes significantly to filling the knowledge gap regarding breastfeeding practices and the determinants of EBF in Romania. It is worth noting that before this study, the last survey that evaluated breastfeeding practices in Romania was conducted in 2011.

## 5. Conclusions

The findings of the present study suggest that during the last decade, in Romania, there has been a positive evolution in the rates of all breastfeeding practices assessed. These improving rates point toward the continuation of efforts to develop effective national policies and programs for the promotion, protection, and support of breastfeeding. Particular emphasis should be given to the creation in maternity hospitals of an environment that is supportive towards breastfeeding.

## Figures and Tables

**Table 1:** Sociodemographic and pre/perinatal characteristics of the participants (*n* = 1399).

Characteristics	Frequency	Percent
**Sociodemographic Characteristics**		
**Maternal age (years)**		
<18	31	2.2
18–24	184	13.2
25–34	940	67.2
≥35	244	17.4
**Place of residence**		
Urban	1027	73.4
Rural	372	26.6
**Marital status**		
Married/Living with a partner	1382	98.8
Single/Divorced/Widowed	17	1.2
**Education**		
≤Secondary school	118	8.4
Completed high school or equivalent	304	21.7
Bachelor’s degree or higher	977	69.9
**Occupation**		
Employed	1231	88.0
Unemployed	168	12.0
**Family financial wellbeing ^#^**		
Poor	235	16.8
Fair	301	21.5
Good	309	22.1
Very good	172	12.3
Excellent	180	12.9
**Parity**		
Primiparous	833	59.5
Multiparous	566	40.5
**Child gender**		
Female	683	48.8
Male	716	51.2
**Child age at interview (months)**		
0–5	377	26.9
6–11	405	29.0
12–23	617	44.1
**Duration of parental leave**		
Less than 22 months from childbirth	268	19.2
22 months or more from childbirth	1131	80.8
**Pre/perinatal characteristics**		
**Gestational age at delivery (weeks)**		
<37	170	12.1
≥37	1229	87.9
**Birth weight (grams)**		
<2500	69	4.9
2500–4199	1280	91.5
≥4200	50	3.6
**Mode of delivery**		
Vaginal delivery	678	48.5
Caesarean section	721	51.5
**Place of delivery**		
Public hospital	1021	73.0
Private hospital	378	27.0
**Skin-to-skin contact the first hour after birth**		
Yes	303	21.7
No	1096	78.3
**Initiation of breastfeeding the first hour after birth**		
Yes	340	24.3
No	1059	75.7
**Prenatal birth and infant care classes**		
Yes	401	28.7
No	998	71.3
**Breastfeeding education during prenatal visits**		
Yes	699	50.0
No	700	50.0
**Postnatal breastfeeding education**		
Yes	1053	75.3
No	346	24.7

^#^ 202 of mothers refused to answer the question about family financial wellbeing.

**Table 2:** Breastfeeding practices among the study participants (*n* = 1399).

Breastfeeding Practice	Frequency	Percent
Exclusive breastfeeding under 6 months (EBF) (*n* = 379)	177	46.7
Continued breastfeeding at 1 year (*n* = 203)	110	54.2
Continued breastfeeding at 2 years (*n* = 221)	67	30.3
Children ever breastfed (*n* = 1399)	1339	95.7

**Table 3:** Unadjusted and adjusted odds ratios of sociodemographic and pre/perinatal factors associated with exclusive breastfeeding under 6 months.

Characteristics	Unadjusted OR (95% CI)	*p*-Value	Adjusted OR (95% CI)	*p*-Value
**Sociodemographic Characteristics**				
**Maternal age (years)**				
<18	2.70 (1.07, 6.80)	0.035 *	2.81 (0.98, 8.06)	0.055
18–24	1.00			
25–34	1.19 (0.73, 1.95)	0.487	1.17 (0.68, 2.02)	0.570
≥35	0.81 (0.43, 1.51)	0.503	0.87 (0.43, 1.76)	0.704
**Place of residence**				
Urban	1.00			
Rural	1.07 (0.75, 1.52)	0.725		
**Education**				
≤Secondary school	1.89 (1.01, 3.54)	0.046 *	1.46 (0.71, 3.01)	0.306
Completed high school or equivalent	1.00			
Bachelor’s degree or higher	1.51 (0.98, 2.33)	0.059	1.34 (0.82, 2.21)	0.243
**Occupation**				
Employed	1.00			
Unemployed	1.17 (0.74, 1.87)	0.497		
**Family financial wellbeing**				
Poor	1.00			
Fair	0.60 (0.37, 0.99)	0.044 *	0.63 (0.37, 1.08)	0.094
Good	0.70 (0.44, 1.14)	0.150	0.72 (0.41, 1.25)	0.236
Very good	0.99 (0.59, 1.67)	0.966	0.95 (0.52, 1.77)	0.880
Excellent	0.58 (0.32, 1.03)	0.064	0.57 (0.29, 1.14)	0.110
**Parity**				
Primiparous	1.00			
Multiparous	1.22 (0.89, 1.67)	0.226		
**Child gender**				
Female	1.10 (0.80, 1.50)	0.564		
Male	1.00			
**Duration of parental leave**				
Less than 22 months from childbirth	1.00		1.00	
22 months or more from childbirth	5.71 (2.77, 11.76)	0.000 **	7.90 (3.43, 18.22)	0.000 **
**Pre/perinatal characteristics**				
**Gestational age at delivery (weeks)**				
<37	1.00			
≥37	1.35 (0.79, 2.28)	0.269		
**Birth weight (grams)**				
<2500	0.53 (0.21, 1.34)	0.181		
2500–4199	1.00			
≥4200	1.30 (0.60, 2.81)	0.511		
**Mode of delivery**				
Vaginal delivery	1.37 (1.00, 1.89)	0.050 *	1.27 (0.87, 1.87)	0.218
Caesarean section	1.00		1.00	
**Place of delivery**				
Public hospital	1.00		1.00	
Private hospital	1.55 (1.11, 2.16)	0.011 *	1.62 (1.06, 2.48)	0.026 *
**Skin-to-skin contact the first hour after birth**				
Yes	1.72 (1.21, 2.43)	0.002 *	1.45 (0.94, 2.24)	0.094
No	1.00		1.00	
**Initiation of breastfeeding the first hour after birth**				
Yes	1.53 (1.08, 2.16)	0.015 *	1.14 (0.70, 1.87)	0.601
No	1.00		1.00	
**Prenatal birth and infant care classes**			-	
Yes	1.11 (0.79, 1.56)	0.561	-	
No	1.00		-	
**Breastfeeding education during prenatal visits**				
Yes	1.15 (0.84, 1.58)	0.371	-	
No	1.00		-	
**Postnatal breastfeeding education**				
Yes	1.00		-	
No	1.12 (0.78, 1.60)	0.548	-	

OR: Odds Ratio, CI: Confidence Interval. * Significant at *p* < 0.05, ** significant at *p* < 0.001.
